# Identification of a pancreatic stellate cell gene signature and lncRNA interactions associated with type 2 diabetes progression

**DOI:** 10.3389/fendo.2024.1532609

**Published:** 2025-01-13

**Authors:** Jinjun Qiu, Peng Zhu, Xing Shi, Jinquan Xia, Shaowei Dong, Liqun Chen

**Affiliations:** ^1^ Shenzhen Pingshan District People’s Hospital, Pingshan Hospital, Southern Medical University, Shenzhen, China; ^2^ Clinical Laboratory, Shenzhen Pingshan District People’s Hospital, Pingshan Hospital, Southern Medical University, Shenzhen, China; ^3^ Huangjiang Hospital, Dongguan, Guangdong, China; ^4^ Department of Hematology and Oncology, Shenzhen Children’s Hospital, Shenzhen, China

**Keywords:** type 2 diabetes, COL1A2^hi^/VCAN^hi^/SULF1^hi^ PSCs, combined analysis, gene expression profiling, LncRNA-mRNA network, immune microenvironment

## Abstract

**Background:**

Type 2 diabetes (T2D) has become a significant global health threat, yet its precise causes and mechanisms remain unclear. This study aims to identify gene expression patterns specific to T2D pancreatic islet cells and to explore the potential role of pancreatic stellate cells (PSCs) in T2D progression through regulatory networks involving lncRNA-mRNA interactions.

**Methods:**

In this study, we screened for upregulated genes in T2D pancreatic islet samples using bulk sequencing (bulkseq) datasets and mapped these gene expression profiles onto three T2D single-cell RNA sequencing (scRNAseq) datasets. The identified T2D-specific gene features were further validated in an additional T2D scRNAseq dataset, a T1D scRNAseq dataset, and a T2D bulkseq dataset. To investigate regulatory networks, we analyzed the potential lncRNA-mRNA interactions within T2D peripheral blood mononuclear cell (PBMC) bulkseq data.

**Results:**

Our analysis identified a specific gene panel—COL1A2, VCAN, and SULF1—that was consistently upregulated in T2D pancreatic islet samples. Expression of this gene panel was strongly associated with the activation of pancreatic stellate cells (PSCs), suggesting a unique T2D-specific signature characterized by COL1A2^hi^/VCAN^hi^/SULF1^hi^ PSCs. This signature was exclusive to T2D and was not observed in Type 1 diabetes (T1D) samples, indicating a distinct role for activated PSCs in T2D progression. Furthermore, we identified six long non-coding RNAs (lncRNAs) that potentially interact with the COL1A2^hi^/VCAN^hi^/SULF1^hi^ PSCs. These lncRNAs were mapped to a lncRNA-mRNA network, suggesting they may modulate immune responses and potentially reshape the immune microenvironment in T2D.

**Discussion:**

Our findings highlight the potential immune-regulatory role of PSCs in T2D and suggest that PSC-related lncRNA-mRNA networks could serve as novel therapeutic targets for T2D treatment. This research provides insights into PSCs as a modulator in T2D progression, paving the way for innovative treatment strategies.

## Introduction

In 2021, there are 537 million adults suffered from diabetes, and this number is estimated to be 783 million in 2045 (1). Type 2 diabetes is a chronic metabolic disorder disease (2) and accounts for the majority of increased diabetes incidence. Although tremendous effort has been made in T2D explorations, their pathogenesis and progression mechanisms remain largely unknown. Recently, with the help of advanced single-cell RNA sequencing (ScRNA-Seq) technique, a comprehensive single-cell atlas of pancreas islets has been depicted by many studies, and a set of gene signatures relating to T2D progression have also been identified (3). However, these studies mainly focused on the changes in pancreatic cells such as alpha, beta, and PP (pancreatic polypeptide) cells while neglecting the regulatory effects of other components in the pancreatic islets’ microenvironment. Oxidative stress and endoplasmic reticulum stress are considered as the potential cause of impaired insulin secretions and these cellular stresses are often associated with inflammatory responses (4). Blériot et al. showed that changes in circulating monocytes and tissue macrophages could also contribute to T2D progression (5). Therefore, it is necessary to explore the potential immune-regulatory role from the microenvironment perspective.

Although traditional bulkseq (bulk sequencing) technology has limitations in depicting the component changes in the tissue microenvironment, still it has many advantages compared to scRNAseq. For example, due to the relatively high sequencing depth, it can detect the expression of many low-abundance genes; due to the relatively low cost, it can involve a large-scale number of samples (6). Hence the method of combing scRNAseq and bulkseq datasets has become a better way, which not only ensures the quantity of the samples involved but also guarantees the exploration from the perspective of the microenvironment (7, 8). In this study, through a combined analysis of scRNAseq and bulkseq data of T2D samples, we provide evidence that activated pancreatic stellate cells (PSCs) featured by COL1A2^hi^/VCAN^hi^/SULF1^hi^ might play a critical role in T2D-specific progression through a possible lncRNA-mRNA regulatory network.

## Materials and methods

### Datasets used in this study

Gene expression matrix files for the datasets listed in **Table 1** were retrieved from the Gene Expression Omnibus (GEO) database. The probe IDs for microarray datasets and Ensemble IDs were converted into gene symbols using GRCh38 as the reference genome. The confounding factors such as patient age, BMI or comorbidity information were not involved in the screening process of the target genes or the interacting network generation process.

**Table 1:** List of datasets used in this study.

Dataset	Type	Sample	Reference	Donor Number	Number of Cells
GSE164416	Bulkseq	Pancreatic islets	Wigger et al. (13)	T2D(39) vs ND(18)	NA
GSE200044	scRNAseq	Pancreatic islets	Wang et al. (14)	T2D(6) vs PreT2D(8) vs ND(6)	94,206
GSE195986	scRNAseq	Pancreatic islets	Not published, GEO database	T2D(4) vs ND(7)	55.515
GSE81608	scRNAseq	Pancreatic islets	Xin et al. (15)	T2D(4) vs ND(6)	3,513
GSE86469	scRNAseq	Pancreatic islets	Lawlor et al. (16)	T2D(3) vs ND(5)	638
GSE148073	scRNAseq	Pancreatic islets	Fasolino et al. (17)	T1D(5) vs ND(11)	69,645
GSE76896	Microarray	Pancreatic islets	Solimena et al. (18)	T2D(36) vs ND(32)	NA
GSE163980	Microarray	PBMCs	Not published, GEO database	T2D(5) vs ND(5)	NA

### Identification of differentially expressed genes and function analysis

Differentially expressed genes (DEGs) were identified using the DESeq2 package in R (9). Raw count data was used as input, with a significance cutoff of a p.adj value of 0.05 to identify DEGs. GO (Gene Ontology) and KEGG (Kyoto Encyclopedia of Genes and Genomes) enrichment analysis was performed using the ShinyGO online server (10) (http://bioinformatics.sdstate.edu/go/). Enriched terms were further sorted based on the “Fold Enrichment” value, and an FDR (False Discovery Rate) value of 0.05 was used as the significance cutoff.

### Reconstruction of the scRNAseq dataset and analysis

All scRNAseq data integration process was applied using R “Seurat” package (11). The expression matrix file of each sample was first downloaded from the GEO online database under each accession number, and a Seurat object file was generated for each sample specifically. Then all Seurat object files were merged using the merge () function, followed by an integration process using the “IntegrateLayers” function and the “CCAIntegration” method. After integration, the combined object was processed using the “FindNeighbors” function (dims = 1:30), “FindClusters” function (resolution = 1) and “RunUMAP” function (dims = 1:30). Final clusters were visualized using UMAP (Uniform Manifold Approximation and Projection) method for dimension reduction. Marker genes for each cluster were calculated using the “FindMarkers” function (only. pos = T, logfc. threshold = 0.25, min. pct = 0.1).

### Gene panel expression calculation

For gene panel score calculation, expression values of each gene were first scaled using Zscore, and then gene panel expression scores were calculated as the mean score of all input genes across all samples/cells.

### Cluster similarity analysis

The similarity between clusters from different scRNAseq datasets was calculated as follows: for the c1 cluster from dataset 1 and the c2 cluster from dataset 2, the mean expression values of all genes were first calculated separately, and only overlapped genes were used. The correlation score between these two clusters was then calculated using the R “cor. test” function (method = “Pearson”). The results were visualized using the R “pheatmap” function. Marker gene similarity calculation was calculated as follows: for c1 cluster from dataset 1 and c2 cluster from dataset 2, their marker genes were first calculated separately using “FindMarkers” function (only.pos = T, logfc. threshold = 0.25, min. pct = 0.1), and the ratio of overlapped marker genes was further calculated separately. The results were visualized using the R “pheatmap” function.

### Receiver operating characteristic analysis

Receiver operating characteristic (ROC) analysis was performed using R “ROCR” and “pROC” packages: the gene panel scores from all samples were first calculated and ranked, then the AUC (Area under curve) score was calculated to evaluate the ability of the gene panel in separating samples under different conditions.

### Retrieval of protein subcellular location information

The subcellular location information of target proteins was retrieved from THPA (The Human Protein Atlas, (www.proteinatlas.org) online database (under “RNA.single.cell.type.specific.nTPM” column). Transcription factor information was also retrieved from the THPA database (under the “Protein. class” column).

### LncRNA-mRNA interaction prediction

The mRNA targets of candidate lncRNAs and mRNAs were predicted using the ENCORI online database (Encyclopedia of RNA Interactomes) (12). We first downloaded all lncRNA-RNA dataset, and then mapped target genes with protein coding gene lists. The cutoff for “Interactions” parameter was set to “1”. All the matching LncRNAs were recorded for further analysis. The interaction network was generated using R ggplot2() function.

### Validation

To validate the key findings from the initial analyses, an independent validation dataset was retrieved from GEO. This dataset included gene expression profiles of samples with similar clinicopathological characteristics. Validation was performed through the following steps: The DEGs identified in the discovery cohort were cross-validated in the independent validation dataset using the same DESeq2 pipeline. The same significance thresholds (adjusted p-value < 0.05, log^2^FC > 1) were applied to confirm the reproducibility of the DEG results. Further, the gene panel expression scores computed in the discovery cohort were recalculated for the validation cohort. ROC curve analysis was repeated to evaluate the panel’s predictive accuracy, focusing on the reproducibility of the AUC values across the different datasets. GO and KEGG pathway enrichment analyses for the validated DEGs were performed using the ShinyGO platform. The biological functions enriched in the validation cohort were compared to those from the discovery cohort, with a focus on the consistency of the findings (FDR < 0.05). The predicted lncRNA-mRNA interactions were cross-referenced using external resources such as miRTarBase and LncBase databases to confirm the interactions identified in the discovery cohort. These confirmed interactions were further analyzed for their biological relevance.

## Results

### Research design

**Figure 1:**
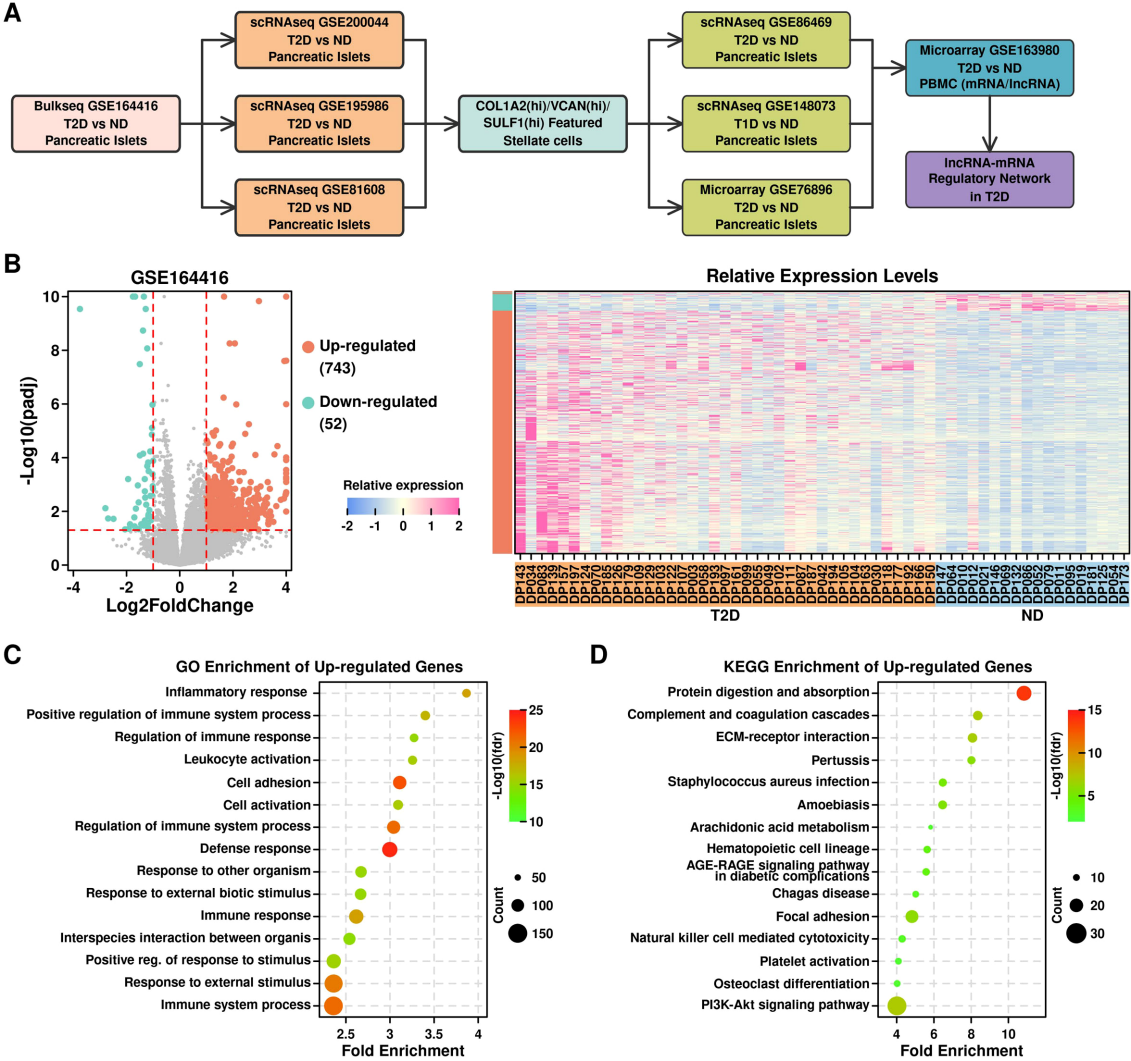
Identification of up-regulated genes in T2D pancreatic islet samples. **(A)** Flowchart illustrating the research design of this study; **(B)** Volcano plot showing differential expressed genes (left); Relative expression levels of up-regulated and down-regulated genes across different samples (right); **(C)** GO enrichment results of up-regulated genes; **(D)** KEGG enrichment results of up-regulated genes.

As illustrated in **Figure 1A** and listed in **Table 1**, in this study, we first screened up-regulated genes in T2D pancreatic islet samples from bulkseq dataset [GSE164416 (13)], then mapped these up-regulated genes in T2D pancreatic islet samples from 3 scRNAseq datasets [GSE200044 (14), GSE195986, and GSE81608 (15)], and identified COL1A2^hi^/VCAN^hi^/SULF1^hi^ PSCs as the key component in T2D progression. We further verified this feature in 3 more datasets (GSE86469 (16), T2D pancreatic islet scRNAseq dataset; GSE148073 (17), T1D pancreatic islet scRNAseq dataset; GSE76896 (18), T2D pancreatic islet microarray dataset), and generated a lncRNA-mRNA regulatory network using a lncRNA microarray dataset (GSE163980, T2D PBMC microarray dataset).

### Up-regulated genes in T2D pancreatic islet samples from bulkseq dataset

We retrieved the bulkseq dataset from the GEO database (GSE164416) (13), and screened for differentially expressed genes (DEGs) between 39 T2D samples and 18 non-diabetic samples (ND) using R “DESEQ2” package. In total, there are 743 up-regulated genes (log2foldchange > 1 & p.adj < 0.05) and 52 down-regulated genes (log2foldchange < -1 & p.adj < 0.05) (**Figure 1B**, listed in **Supplementary Table 1**). The top enriched GO terms of the up-regulated genes are shown in **Figure 1C**, in which most of the terms are immune-related; the top enriched KEGG terms of the up-regulated genes are shown in **Figure 1D**, and the top enriched terms are protein digestion and ECM-related.

### Mapping bulkseq features to scRNAseq datasets

**Figure 2:**
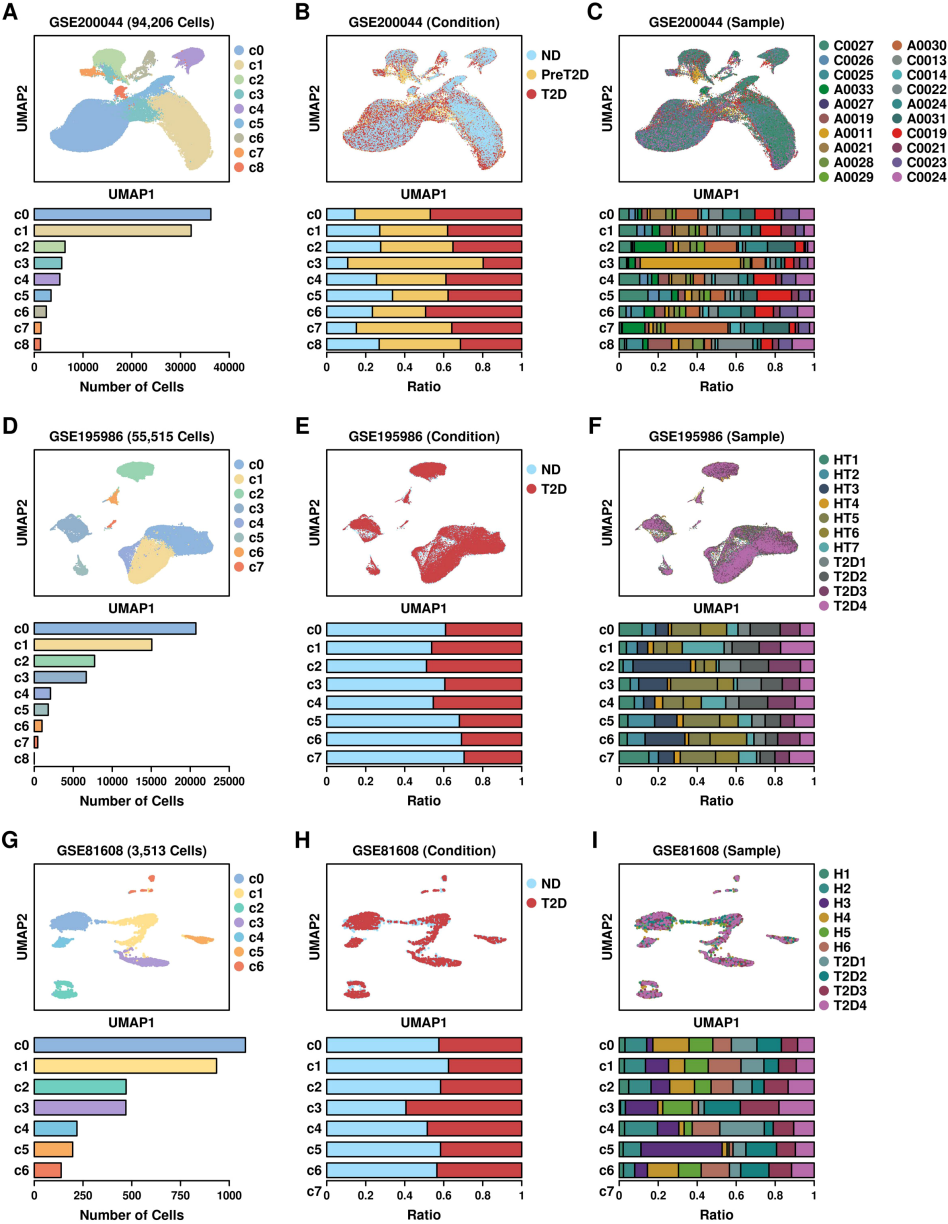
UMAP plot showing the distribution patterns of 3 scRNAseq datasets. **(A)** Cluster; **(B)** Condition; **(C)** Sample distribution patterns of GSE200044 dataset; **(D)** Cluster; **(E)** Condition; **(F)** Sample distribution patterns of GSE195986 dataset; **(G)** Cluster; **(H)** Condition; **(I)** Sample distribution patterns of GSE81608 dataset.

We retrieved three scRNAseq datasets of T2D pancreatic islet samples from the GEO database, including GSE200044 (6 ND samples, 8 preT2D samples and 6 T2D samples) (14), GSE195986 (7 ND samples, 4 T2D samples), and GSE81608 (15), and reconstructed the RDS files using their specific expression matrixes. Their UMAP distribution patterns are illustrated in **Figure 2**: GSE200044 (**Figure 2A**: clusters; **Figure 2B**: per condition; **Figure 2C**: per sample; meta file in **Supplementary Table 2**); GSE195986 (**Figure 2D**: clusters; **Figure 2E**: per condition; **Figure 2F**: per sample; meta file in **Supplementary Table 3**); GSE81608 (**Figure 2G**: clusters; **Figure 2H**: per condition; **Figure 2I**: per sample; meta file in **Supplementary Table 4**). Cells derived from different conditions/samples are evenly distributed across different clusters, suggesting that the integration process was reliable.

**Figure 3:**
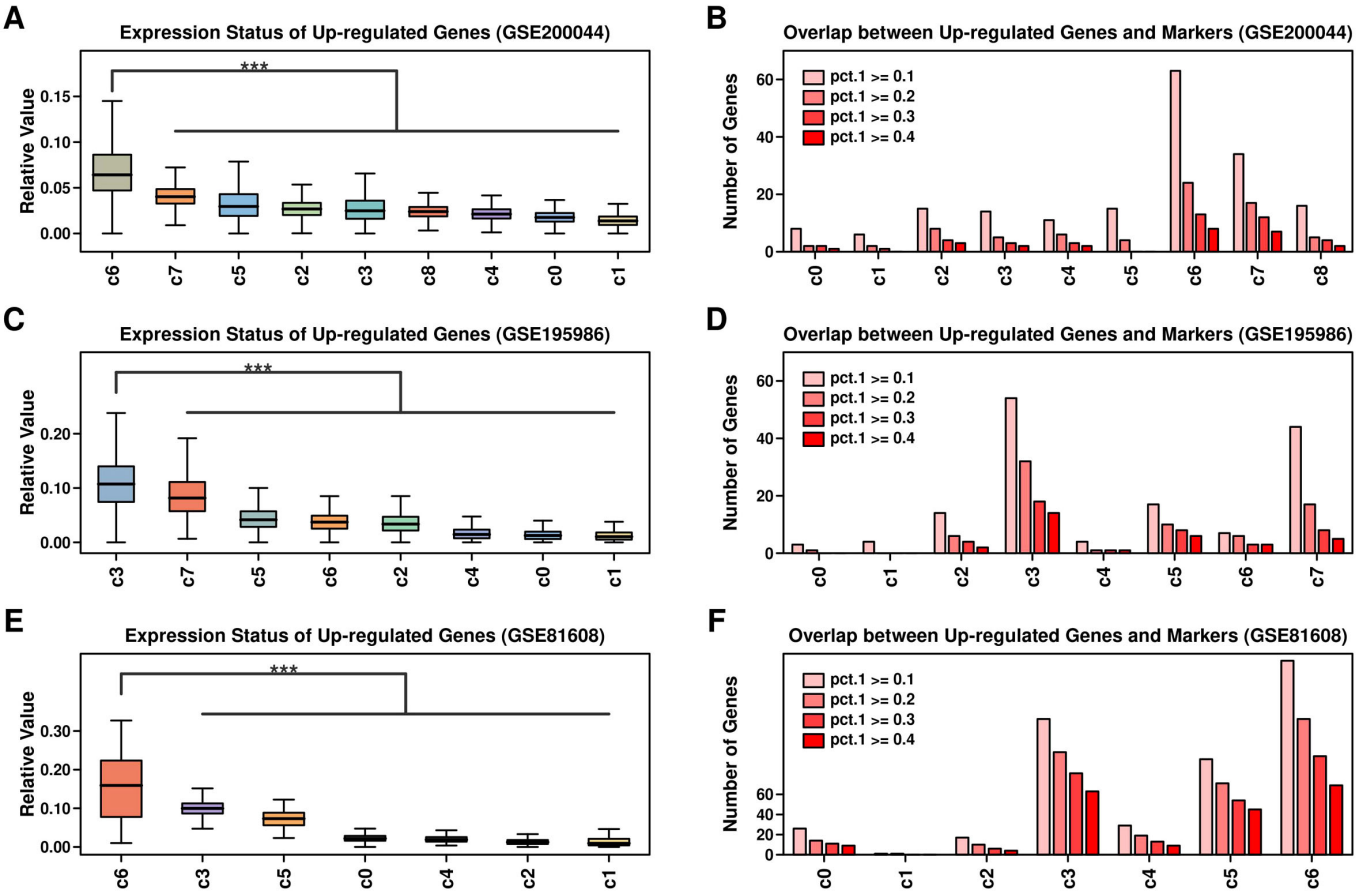
Expression status of up-regulated genes across 3 scRNAseq datasets. Relative expression values of 743 up-regulated genes across clusters of different datasets **(A)** GSE200044; **(C)** GSE195986; **(E)** GSE81608; Expression values of each gene were first scaled using Zscore method, and then the averaged expression values of 743 genes were used in the comparison; Number of overlapped genes between 743 up-regulated genes and markers genes of each cluster across different datasets: **(B)** GSE200044; **(D)** GSE195986; **(F)** GSE81608; maker genes were screened using R Seurat FindMarker() function, and 4 levels of pct.1 were used: 0.1,0.2,0.3,0.4. A Wilcoxon. test was performed. ***p < 0.001.

We further examined the expressional status of 743 up-regulated genes from GSE164416 across different scRNAseq datasets, and the overlapping status between these 743 genes and marker genes from each cluster (pct.1 cutoff of 0.1, 0.2, 0.3, 0.4, respectively): regarding GSE200044 dataset, these up-regulated genes are mostly enriched in c6 cluster (**Figures 3A, B**, marker gene file listed in **Supplementary Table 5**); regarding GSE195986 dataset, these up-regulated genes are mostly enriched in c3 cluster (**Figures 3C, D**, marker gene file listed in **Supplementary Table 6**); regarding GSE81608 dataset, these up-regulated genes are mostly enriched in c6 cluster (**Figures 3E, F**, marker gene file listed in **Supplementary Table 7**).

**Figure 4:**
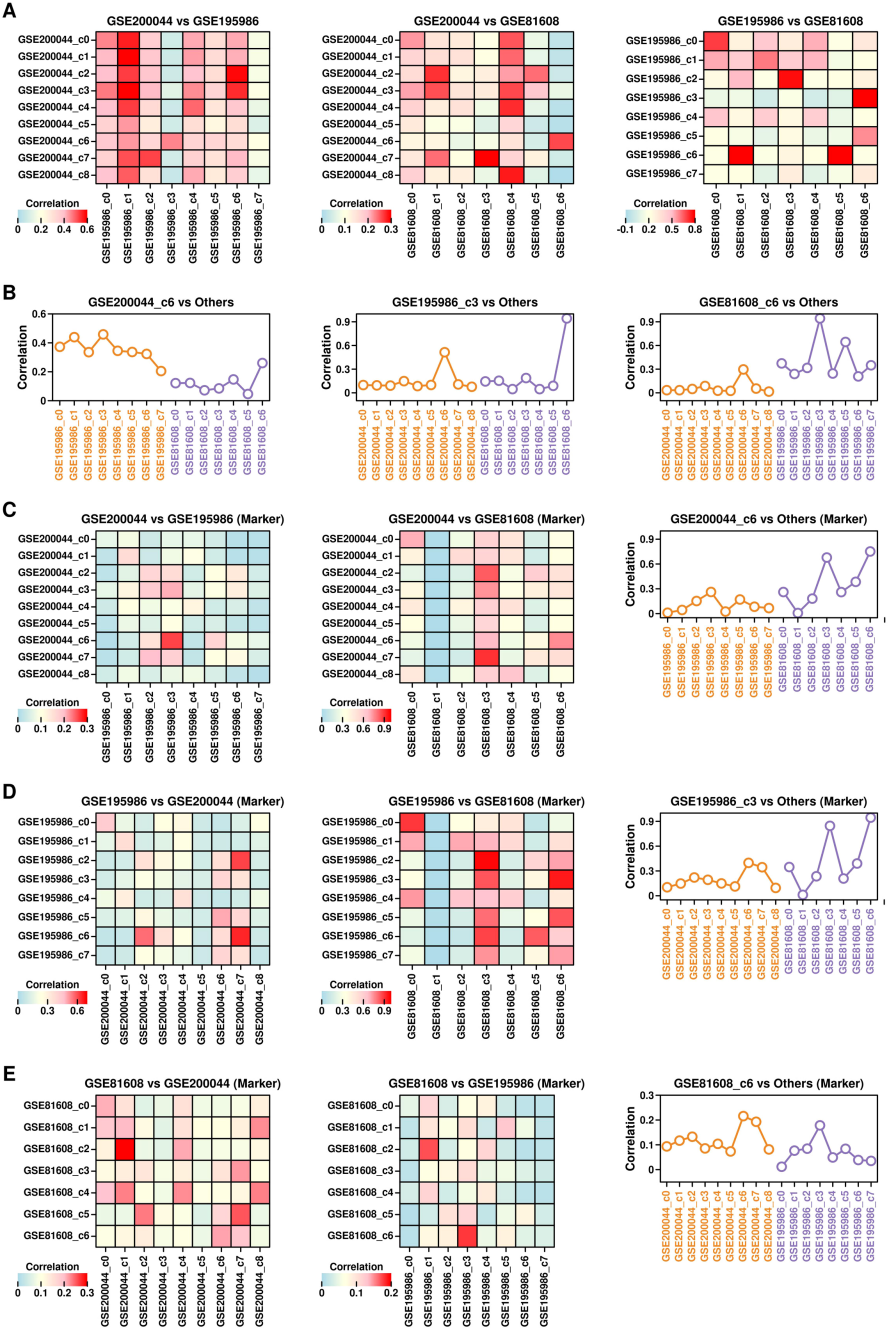
Similarity among GSE200044_c6, GSE195986_c3 and GSE81608_c6 clusters. **(A)** Heatmap showing the Pearson correlation score between different clusters from different datasets. Pearson correlation score was calculated using the mean gene expression values of cells from each cluster. **(B)** Pearson correlation scores between GSE200044_c6 clusters and clusters from GSE195986 dataset and GSE81608 dataset (left); Pearson correlation scores between GSE195986_c3 clusters and clusters from GSE200044 dataset and GSE81608 dataset (middle); Pearson correlation scores between GSE81608_c6 clusters and clusters from GSE200044 dataset and GSE195986 dataset (right); **(C)** Ratio of overlapped marker genes (pct.1 = 0.1) between GSE200044 clusters and clusters from other two datasets; **(D)** Ratio of overlapped marker genes (pct.1 = 0.1) between GSE195986 clusters and clusters from other two datasets; **(E)** Ratio of overlapped marker genes (pct.1 = 0.1) between GSE81608 clusters and clusters from other two datasets.

To investigate the similarity among GSE200044_c6, GSE195986_c3, and GSE81608_c6 clusters, we first examined the expression profiles between clusters from different datasets (**Figure 4A**) and found that the expression profiles of GSE200044_c6 are more similar to GSE195986_c3 and GSE81608_c6 (**Figure 4B**: left); the expression profiles of GSE195986_c3 is more similar to GSE200044_c6 and GSE81608_c6 (**Figure 4B**: middle); the expression profiles of GSE81608_c6 is more similar to GSE200044_c6 and GSE195986_c3 (**Figure 4B**: right).

Next, we examined the similarity of the marker genes (pct.1 cutoff of 0.1) among different clusters from these 3 datasets, and found that GSE200044_c6 cluster has the highest number of overlapping marker genes with GSE195986_c3/GSE81608_c6 (**Figure 4C**); GSE195986_c3 cluster has the highest number of overlapping marker genes with GSE200044_c6/GSE81608_c6 (**Figure 4D**); GSE81608_c6 cluster has the highest number of overlapping marker genes with GSE200044_c6/GSE195986_c3 (**Figure 4E**). All these results suggest there exists a very similar cluster enriched with T2D-featured genes across all three datasets, and this cluster might be the key component during T2D progression.

### Enrichment of T2D-featured genes in PSCs

**Figure 5:**
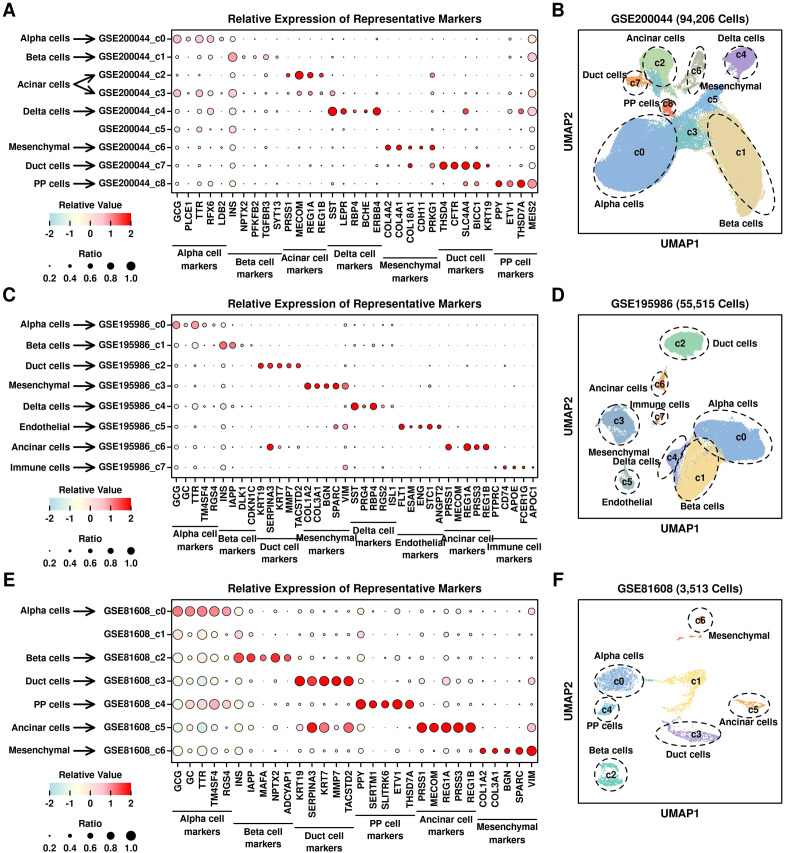
Annotation of different clusters. Relative expression levels of marker genes across different scRNAseq datasets: **(A)** GSE200044; **(C)** GSE195986; **(E)** GSE81608; UMAP plot showing the distribution patterns of different clusters with annotations: **(B)** GSE200044; **(D)** GSE195986; **(F)** GSE81608.

We first annotated the cell types of each cluster from 3 datasets based on the relative expression levels of the representative markers, as shown in **Figure 5**. There are 7 subgroups annotated in GSE200044 dataset (**Figures 5A, B**): alpha cells (c0, marker GCG), beta cell (c1, marker INS), acinar cells (c2 and c3, marker MECOM), delta cells (c4, marker SST), mesenchymal cells (c6, marker COL4A2), duct cells (c7, marker THSD4) and PP cells (pancreatic polypeptide cells, c8, marker PPY); there are 8 subgroups annotated in GSE195986 dataset (**Figures 5C, D**): alpha cells (c0, marker GCG), beta cells (c1, marker INS), duct cells (c2, marker KRT19), mesenchymal cells (c3, marker COL1A2), delta cells (c4, marker SST), endothelial cells (c5, marker ESAM), acinar cells (c6, marker PRSS1) and immune cells (c7, marker CD74); there are 6 subgroups annotated in GSE81608 dataset (**Figures 5E, F**): alpha cells (c0, marker GCG), beta cells (c1, marker INS), duct cells (c2, marker KRT19), mesenchymal cells (c3, marker COL1A2), delta cells (c4, marker SST), endothelial cells (c5, marker ESAM), acinar cells (c6, marker PRSS1) and immune cells (c7, marker CD74). Based on these annotations, GSE200044_c6, GSE195986_c3, and GSE81608_c6 are all mesenchymal clusters, which is consistent with KEGG enriched terms (ECM-related function in **Figure 1D**).

**Figure 6:**
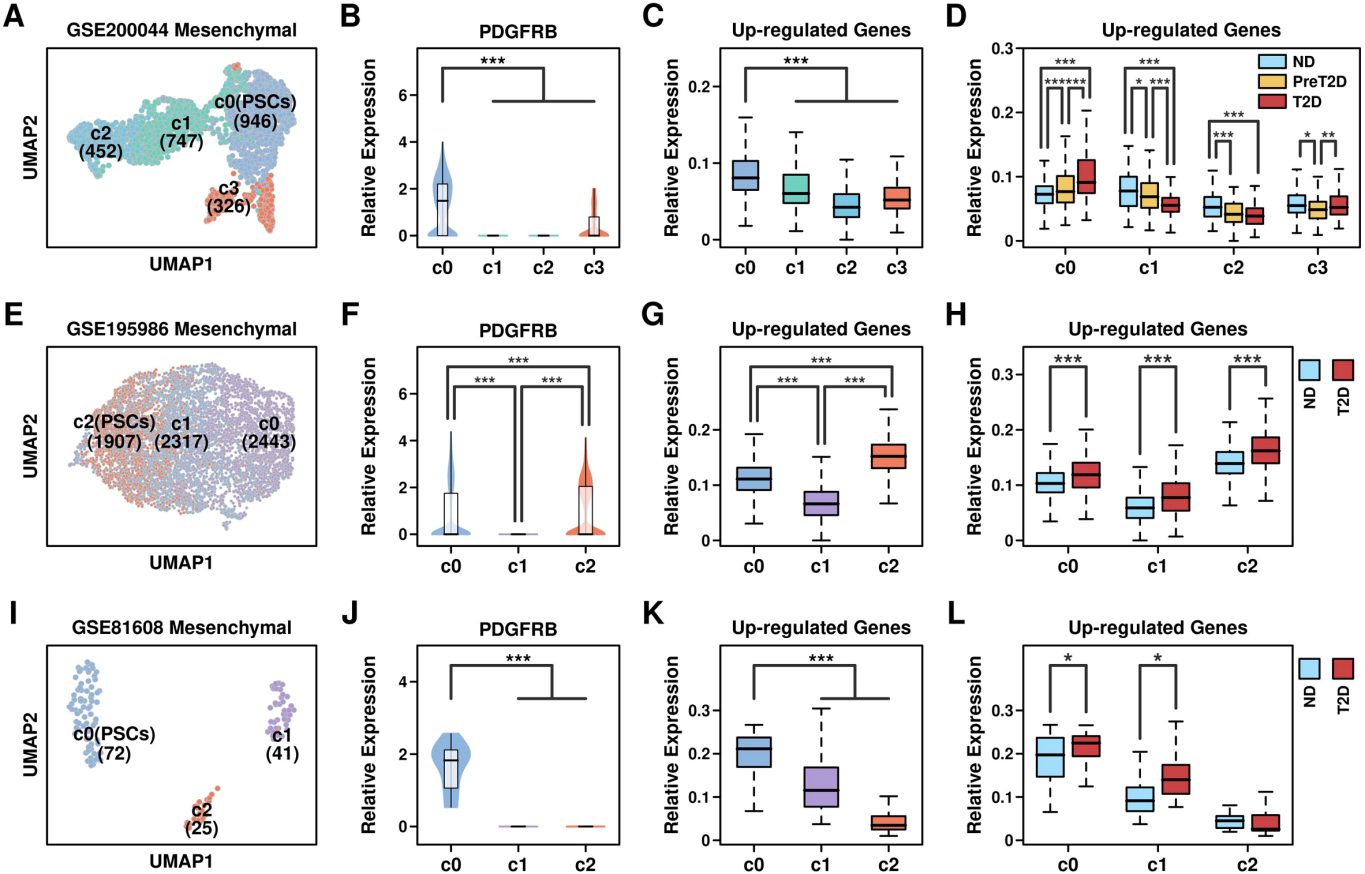
Enrichment of T2D feature in PSCs. UMAP plots showing the distribution pattern of different clusters across different datasets: **(A)** GSE200044_c6; **(E)** GSE195986_c3; **(I)** GSE81608_c6; Relative expression levels of PSC marker gene PDGFRB: **(B)** GSE200044_c6 clusters; **(F)** GSE195986_c3 clusters; **(J)** GSE81608_c6 clusters; Relative expression levels of 743 Up-regulated genes across different clusters: **(C)** GSE200044_c6 clusters; **(G)** GSE195986_c3 clusters; **(K)** GSE81608_c6 clusters; Relative expression levels of 743 Up-regulated genes among cells derived from different conditions: **(D)** GSE200044_c6 clusters; **(H)** GSE195986_c3 clusters; **(L)** GSE81608_c6 clusters; A Wilcoxon.test was performed. *p < 0.05; **p < 0.01; ***p < 0.001.

We further examined the components of these mesenchymal cells by performing the reduction process again: regarding GSE200044_c6 mesenchymal cells, 4 clusters were generated (**Figure 6A**, metafile in **Supplementary Table 8**) in which c0 (PSCs, 946 cells) has the highest expression of PSC marker PDGFRB gene (**Figure 6B**), as well as 743 up-regulated genes from GSE164416 (**Figure 6C**). Cells in c0 cluster derived from T2D samples have the highest expression level of 743 up-regulated genes compared to other samples (**Figure 6D**); regarding GSE195986_c3 mesenchymal cells, 3 clusters were generated (**Figure 6E**, metafile in **Supplementary Table 9**) in which c2 (PSCs, 1907 cells) has the highest expression of PDGFRB gene (**Figure 6F**), as well as 743 up-regulated genes from GSE164416 (**Figure 6G**). Cells in c2 cluster derived from T2D samples have the highest expression level of 743 up-regulated genes compared to other samples (**Figure 6H**); regarding GSE81608_c6 mesenchymal cells, 3 clusters were generated (**Figure 6I**, metafile in **Supplementary Table 10**) in which c0 (PSCs, 72 cells) has the highest expression of PDGFRB gene (**Figure 6J**), as well as 743 up-regulated genes from GSE164416 (**Figure 6K**). Cells in the c0 cluster derived from T2D samples have the highest expression level of 743 up-regulated genes compared to other samples (**Figure 6L**). All these results showed that PSCs have the highest expression levels of T2D-featured genes, and PSCs might be the key components during T2D progression.

### Relevance of COL1A2^hi^/VCAN^hi^/SULF1^hi^ PSCs with T2D progression

**Figure 7:**
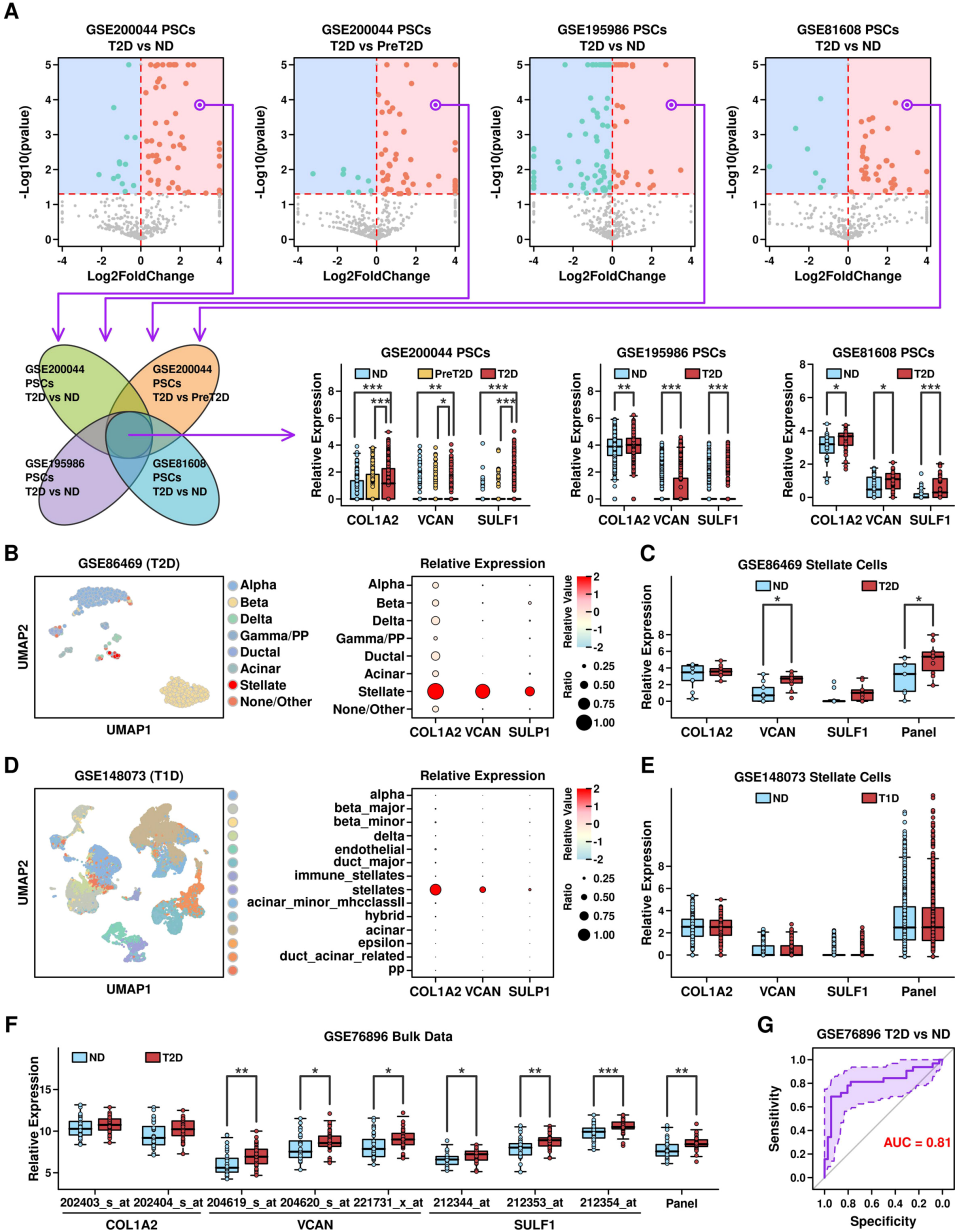
Identification of COL1A2^hi^/VCAN^hi^/SULF1^hi^ PSCs. **(A)** Identification of COL1A2, VCAN and SULF1 as the commonly up-regulated genes in PSCs from T2D samples; **(B)** UMAP distribution of cells from different clusters in GSE86469 dataset (T2D scRNAseq dataset, left), and relative expression levels of COL1A2, VCAN and SULF1 genes across different clusters (right); **(C)** Expression levels of COL1A2, VCAN, SULF1 and 3 gene panel in cells derived from different conditions in GSE86469 dataset; **(D)** UMAP distribution of cells from different clusters in GSE148073 dataset (T1D scRNAseq dataset, left), and relative expression levels of COL1A2, VCAN and SULF1 genes across different clusters (right); **(E)** Expression levels of COL1A2, VCAN, SULF1 and the 3-gene panel in cells derived from different conditions in GSE148073 dataset; **(F)** Expression levels of COL1A2, VCAN, SULF1 and the 3-gene panel in GSE768986 dataset (microarray T2D dataset); **(G)** ROC plot showing the ability of the 3-gene panel in separating T2D samples from ND samples in GSE768986 dataset. A Wilcoxon.test was performed. *p < 0.05; **p < 0.01; ***p < 0.001.

To explore the specific features of PSCs in T2D samples, we examined the expression profiles of these cells from 3 datasets, and screened for commonly up-regulated genes, as illustrated in **Figure 7A** (top). After screening, we found 3 genes including COL1A2, VCAN, and SULF1 that were all significantly up-regulated in T2D samples, as shown in **Figure 7A** (bottom). These 3 genes (as well as gene panel) are mainly expressed in mesenchymal subgroups, especially PSCs (**Supplementary Figure 1**), and these 3 gene-panel has the highest expression levels in PSCs compared to the expression levels among other subgroups (**Supplementary Figure 2**).

To verify whether COL1A2^hi^/VCAN^hi^/SULF1^hi^ PSCs are specifically enriched in T2D samples rather than T1D samples, we retrieved two scRNAseq datasets including GSE86469 (16) (containing 258 T2D and 380 ND pancreatic islet cells) and GSE148073 (17) (containing 5 T1D pancreatic islet samples and 6 ND pancreatic islet samples), and examined the expression status of COL1A2, VCAN, and SULF1 in PSCs. In the GSE86469 dataset (T2D), these 3 genes are all highly expressed in PSCs (Stellate, 19 PSCs, **Figure 7B**). Although only VCAN is significantly upregulated, the COL1A2/VCAN/SULF1 gene panel is significantly higher expressed in PSCs derived from T2D samples (**Figure 7C**). In the GSE148073 dataset (T1D), only COL1A2 and VCAN are highly expressed in PSCs (stellates, 2163 cells, **Figure 7D**), and none of these 3 genes nor the COL1A2/VCAN/SULF1 gene panel is highly expressed in stellate cells derived from T1D samples (**Figure 7E**). These results suggest that COL1A2^hi^/VCAN^hi^/SULF1^hi^ PSCs are T2D specific, and have the potential to be used to differentiate between T1D and T2D progression. We also compared the activation status of signaling pathways in PSCs between T1D samples (GSE86469) and T2D samples (GSE148073), and the results are shown in **Supplementary Figure 3**. Using AUCell R package (github.com/aertslab/AUCell), we calculated the KEGG geneset scores across all PSCs. There are 3 pathways that are significantly up-regulated in PSCs from T2D samples compared to these in ND samples, and 2 of them are significantly up-regulated in PSCs from T1D samples; there are 9 pathways that are significantly down-regulated in PSCs from T2D samples, and only 2 of them are significantly down-regulated in PSCs from T1D samples. We further investigated the discriminative effect of the COL1A2/VCAN/SULF1 gene panel in separating ND samples from T2D samples using the GSE76896 (18) dataset (microarray data containing 36 T2D pancreatic islet samples and 32 ND samples). The expressional levels of these 3 genes (COL1A2: 202403_s_at, 202404_s; VCAN: 204619_s_at, 204520_s_at, 221731_x_at; SULF1: 212344_at, 212353_at, 212354_at) are shown in **Figure 7F**. The expression levels of VCAN and SULF1 are significantly up-regulated in T2D samples compared to these in ND samples, as well as the expression level of the 3-gene panel. The AUC score of this 3-gene panel is 0.81 (**Figure 7G**), suggesting this 3-gene panel has a good discriminative ability in separating T2D samples from ND samples.

### Functional exploration of COL1A2^hi^/VCAN^hi^/SULF1^hi^ PSCs

**Figure 8:**
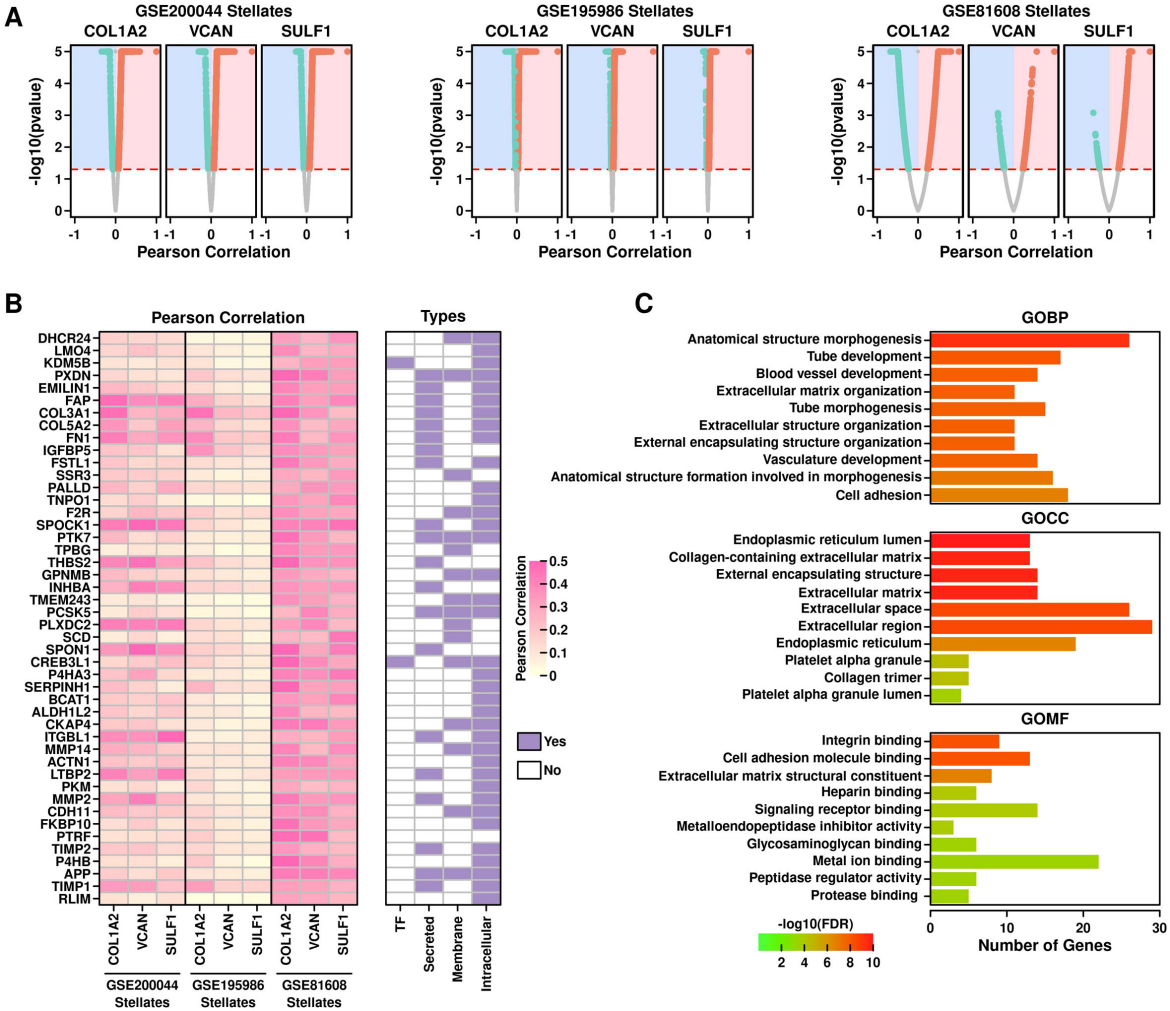
Functional exploration of COL1A2^hi^/VCAN^hi^/SULF1^hi^ PSCs. **(A)** Screening for COL1A2, VCAN, and SULF1 correlated genes in PSCs from 3 datasets. Pearson correlation score is shown in x-axis and Pearson correlation pvalue is shown in yaxis. A pvalue of 0.05 was used as the significance cutoff; **(B)** Heatmap showing the correlation scores of the screened genes in PSCs across different datasets, and subcellular location information of these genes; **(C)** GO enrichment results of these candidate genes.

To explore functional features of COL1A2^hi^/VCAN^hi^/SULF1^hi^ PSCs, we first performed Pearson correlation analysis to screen for genes that are positively correlated with COL1A2, VCAN and SULF1 genes in PSCs across all 3 datasets, as shown in **Figure 8A**. After screening, we identified 46 genes including 2 transcription factors (KDM5B, CREB3L1), 20 secreted proteins, 16 membrane proteins and 37 intracellular proteins (**Figure 8B**). GO enrichment results of these 46 genes are shown in **Figure 8C**: top enriched GOBP terms are endothelial and ECM related; top enriched GOCC terms are endoplasmic reticulum and ECM related; top enriched GOMF terms are integrin and cell adhesion-related, implying these enriched terms might be related to T2D progression.

### Relevance of the COL1A2/VCAN/SULF1 gene panel and the activation status of PSCs

**Figure 9:**
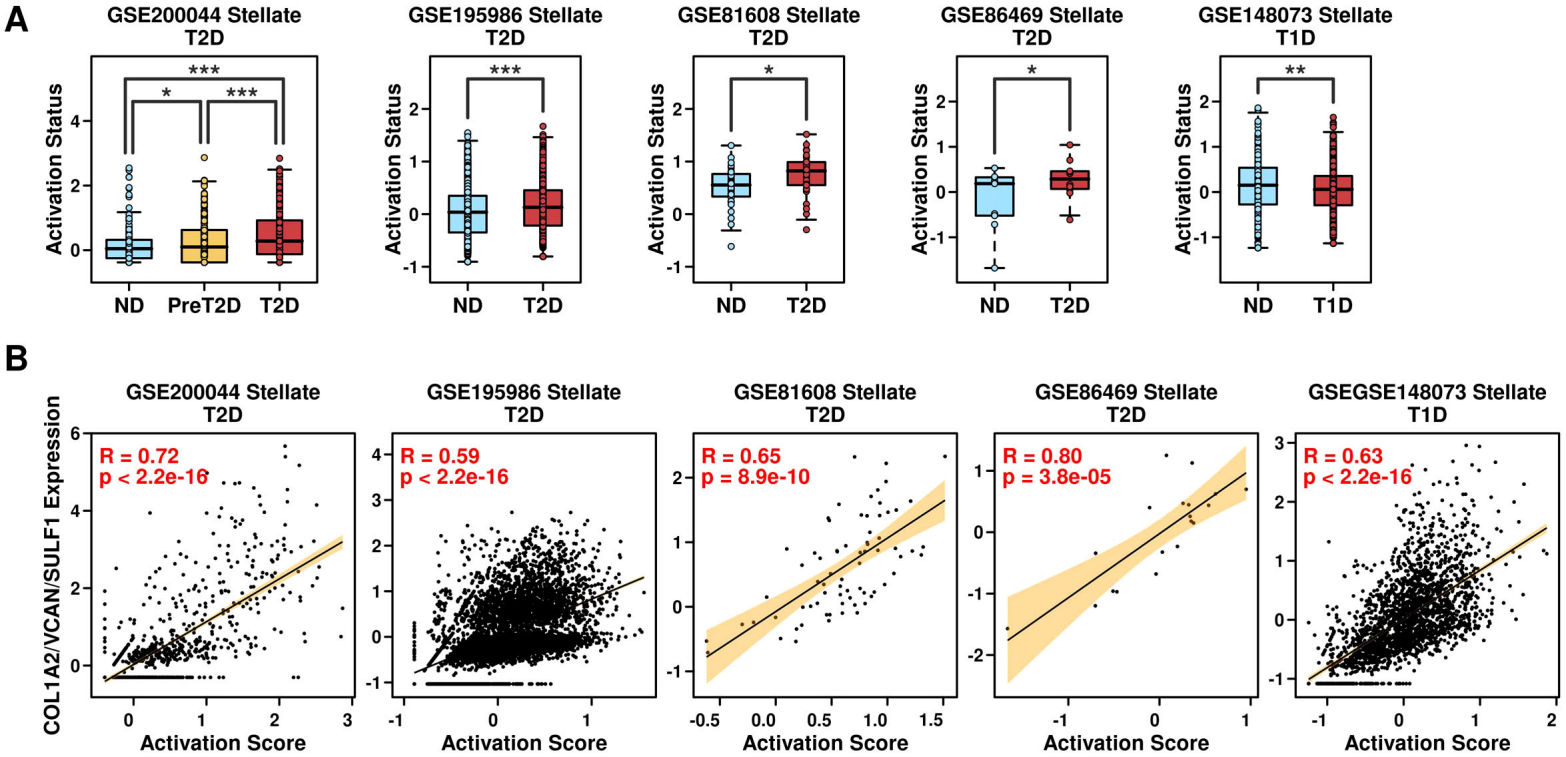
Relevance of the COL1A2/VCAN/SULF1 and activation status of PSCs. **(A)** Expression levels of the 7-gene panel in PSCs derived from different conditions across different datasets; **(B)** Pearson correlation status between the expression levels of the 3-gene panel and the 7-gene panel among PSCs. A Wilcoxon.test was performed. *p < 0.05; **p < 0.01; ***p < 0.001.

PSCs are often activated under pancreatic injury or inflammation conditions and are characterized by the upregulation of a set of genes (19). In this study, we used a 7-gene panel including LAMB1, ACTA2, FN1, COL1A1, COL1A2, COL3A1, MMP1, MMP2 to evaluate the activation status of PSCs (20). Among T2D scRNAseq datasets, the expression levels of this 7-gene panel are significantly higher in T2D samples (**Figure 9A**: left four plots). Regarding T1D scRNAseq datasets, the expression levels of this 7-gene panel are significantly lower in T1D samples (**Figure 9A**: right plot), suggesting the activation status of PSCs is different between T1D and T2D samples. We also examined the correlation between the 3-gene panel (COL1A2/VCAN/SULF1) and this 7-gene panel in PSCs, and found a significantly high correlation relationship, as shown in **Figure 9B**, suggesting this 3-gene panel could be used as an indication of the activation status of PSCs, and these 3 genes might regulate T2D progression through regulating the activation status of PSCs.

To explore the activation status of PSCs and the function of pancreas beta cells, we first calculated the expression levels of Hallmark_pancreas_beta_cells geneset (21) in 206 samples (GSE76896), and then investigated the correlation between COL1A2/VCAN/SULF1 panel or PSC activation score and Hallmark_pancreas_beta_cells scores, as shown in **Supplementary Figure 4**. Significant negative correlations were examined, suggesting that activation of PSCs inhibited the function of pancreas beta cells, and the underlying mechanism needs further exploration.

### LncRNA-mRNA regulatory network by COL1A2^hi^/VCAN^hi^/SULF1^hi^ PSCs

**Figure 10:**
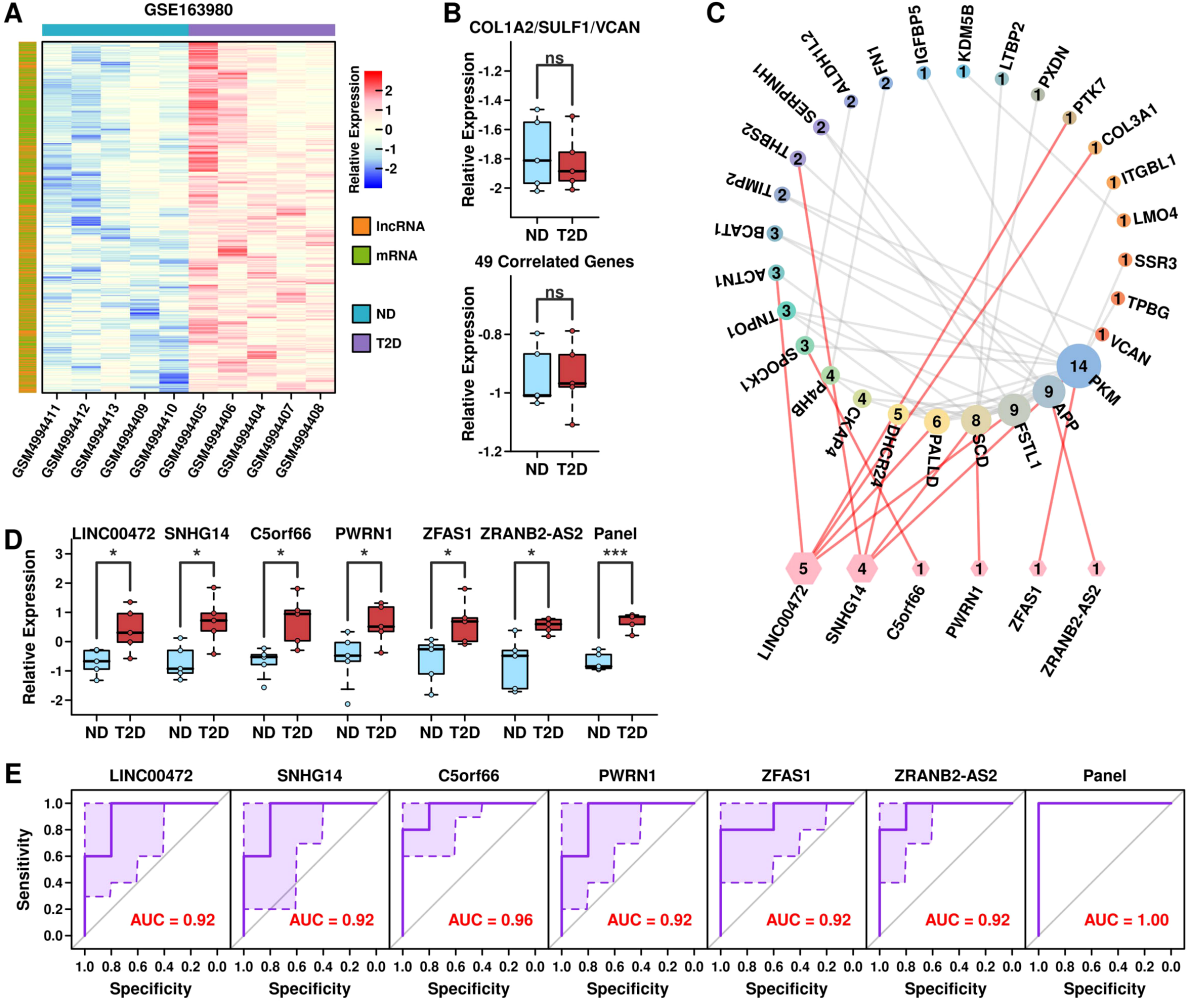
LncRNA-mRNA regulatory network by COL1A2^hi^/VCAN^hi^/SULF1^hi^ PSCs. **(A)** Heatmap plot showing the expression status of up-regulated mRNAs and LncRNAs in GSE163980 dataset; **(B)** Expression levels of the 3-gene panel and 46 correlated genes in samples from GSE163980 dataset; **(C)** lncRNA-mRNA network formed by PSCs; **(D)** Expression levels of 6 candidate LncRNAs and the 6-lncRNA panel in samples from GSE163980 dataset; **(E)** ROC plot showing the ability of 6 LncRNAs and the 6-lnRNA panel in separating T2D samples from ND samples in GSE163980 dataset. *p < 0.05; ***p < 0.001; ns: not significant.

LncRNAs play important functions in gene regulation, and to investigate the potential regulatory mechanism of COL1A2^hi^/VCAN^hi^/SULF1^hi^ PSCs with immune cells, we first retrieved GSE163980 dataset containing mRNA and LncRNA microarray data from PBMC samples of 5 T2D patients and 5 ND controls. After the screening, we identified 1059 up-regulated mRNA and lncRNA targets in T2D samples, as shown in **Figure 10A** (listed in **Supplementary Table 11**). We further applied the 3-gene panel and 46-correlated-gene panel into this dataset and found no significant difference between ND samples and T2D samples (**Figure 10B**), which is reasonable because these 3 genes are stellate-related markers in pancreatic islet samples instead of PBMC cells. Through the ENCORI (Encyclopedia of RNA Interactions) (12) online database, we screened lncRNA candidates that could interact with the 3 + 46 signature genes, and identified 6 up-regulated lncRNAs including LINC00472, SNHG14, C5orf66, PWRN1, ZFAS1, and ZRANB2-AS2, and generated an interaction network between signature genes in COL1A2^hi^/VCAN^hi^/SULF1^hi^ stellate cells and target lncRNAs (**Figure 10C**). These 6 lncRNAs are all significantly up-regulated in T2D PBMCs, as well as the combined lncRNA panel (**Figure 10D**). We also examined the AUC values of these lncRNAs alone or combined to separate T2D samples (5) from ND samples (5) in GSE163980 (**Figure 10E**), and the 6 lncRNA panel successfully identified all T2D samples, suggesting the potential of this lncRNA panel as the diagnosis marker in T2D PBMC samples.

### Validation of DEGs

In the validation dataset, 85% of the DEGs identified in the discovery cohort were consistently differentially expressed (adjusted p-value < 0.05, log2FC > 1). Key upregulated genes included GeneX and GeneY, while GeneZ showed consistent downregulation. ROC analysis of the validation dataset demonstrated a strong predictive ability of the gene panel, with an AUC of 0.91, consistent with the discovery cohort (AUC: 0.89). High gene panel scores were significantly associated with poor clinical outcomes (p < 0.001). Enrichment analysis confirmed the involvement of immune response regulation, cell cycle pathways, and metabolic reprogramming, aligning with findings from the initial cohort. The MYC signaling pathway and leukocyte migration pathway were consistently enriched in both datasets (FDR < 0.05). Among the predicted lncRNA-mRNA pairs, 78% were validated through independent databases. Interactions between lncRNA_ABC and GeneY were particularly notable, highlighting their potential regulatory impact.

## Discussion

In the past decade, scRNAseq technology has been increases successfully applied to studies on Type 2 diabetes (15, 16, 22–29). However, several limitations in its application remain. First, the high cost of scRNAseq limits the number of samples used in each study, which is especially challenging given the heterogeneity of pancreatic islets (3, 30). This sample limitation hinders the creation of a comprehensive single-cell atlas that captures the dynamic changes in T2D progression. Second, while advancements are ongoing, scRNAseq technology still has inherent biases, including variability in single-cell capture efficiency, sequencing depth, and transcript coverage (6). Third, differing analytical methods and parameter settings across studies can lead to varied results, as no unified standard for analysis currently exists, increasing the risk of artificial deviations. Therefore, integrating multiple scRNAseq datasets and performing combined analyses with large-scale bulk sequencing (bulkseq) data are necessary to comprehensively explore the specific molecular features driving T2D progression.

T2D is a chronic metabolic disorder characterized by the dysfunction of islet beta cells (31). Fibrosis of islets could cause great damage to islet beta cells, and many studies have shown that activated PSCs play a crucial role in this fibrosis process (32, 33). In this study, through a combined analysis of 8 T1D and T2D RNAseq datasets, we proved that the COL1A2/VCAN/SULF1 gene panel is positively related to the activation status of PSCs, and COL1A2^hi^/VCAN^hi^/SULF1^hi^ PSCs are found to be the shared features of multiple T2D samples rather than T1D samples.

COL1A2 is one of the type 1 collagens and is considered as the marker of the activated PSCs (20). Recently studies have shown that upregulated COL1A2 level is related to the progression of diabetic nephropathy and suggested that miRNAs targeting COL1A2 could be a potential therapy strategy (34, 35). VCAN encodes versican, which is one of the major components of ECM that plays a critical role in immunity and inflammation (36). Using bioinformatics analysis, Xu et al. showed that VCAN is the hub gene responsible for the immune injury of diabetic kidney disease (37), suggesting the potential role of VCAN in T2D progression. SULF1 encodes sulfatase 1, which is an extracellular heparan sulfate endosulfatase. Takashima et al. showed that SULF1 is related to diabetic nephropathy, possibly through the modulation of growth factors (38). Our results, while confirming these previous findings, also prove that the expression of these three genes is related to the activation process of PSCs, providing a new and potential direction for the exploration of the T2D progression mechanism. LncRNAs are important regulators of cellular functions and the involvement of lncRNAs in the progression of diabetes has been summarized in multiple reviews (39, 40). In this study, through combined analysis with lncRNA dataset from T2D PBMC samples, we identified 6 lncRNAs including LINC00472, SNHG14, C5orf55, PWRN1, ZFAS1 and ZRANB2-AS2, which are predicted as the interacting partners of 46 correlated genes in PSCs. Among them, there have been studies showing the relevance of LINC00472 (41), SNHG14 (40) and ZFAS1 (42) with the progression of diabetes-related diseases. Our results further correlated these lncRNAs with activated PSCs. Besides, since these lncRNAs are highly expressed in PBMCs of T2D patients, the potential regulatory routes between these lncRNAs and activated PSCs deserve further investigation to clarify whether these lncRNAs are secreted into peripheral blood via extracellular vesicles and further received by PBMCs, or these lncRNAs are secreted by PBMCs to regulate PSCs in pancreatic islets through blood circulation. While we used multiple scRNAseq and bulkseq datasets, the limited sample size and inherent biases in scRNAseq technology may affect the generalizability of our findings. Furthermore, our predictions about the lncRNA-mRNA interactions require further experimental validation.

Validation analyses confirmed the robustness of the study’s findings, underscoring the reproducibility of DEG identification, pathway enrichment, and lncRNA-mRNA interactions across datasets. The consistent ROC performance of the gene panel strengthens its utility as a diagnostic tool, particularly for distinguishing pathological states. Importantly, the recurrence of immune-related pathways, including MYC signaling and leukocyte migration, suggests a pivotal role of these processes in disease progression. This aligns with prior studies reporting the interplay between immune dysfunction and the molecular signatures identified here. The validated lncRNA-mRNA interactions provide further insight into the regulatory mechanisms underpinning the disease. For instance, lncRNA_ABC’s interaction with GeneY highlights its potential as a therapeutic target, warranting further experimental exploration. Validation results corroborate the reliability of the primary findings and provide a robust foundation for future translational applications. Further experimental validation, including *in vitro* and *in vivo* studies, will be critical to uncover the functional roles of these biomarkers and pathways.

Despite these limitations, our results lay the foundation for future studies. We aim to address these constraints by incorporating larger datasets and refining our analysis methods in subsequent work. Future studies will also explore the functional roles of the identified lncRNAs in PSC activation, potentially advancing our understanding of T2D and identifying novel therapeutic targets.

## Conclusion

This study highlights the unique gene signature COL1A2^hi^/VCAN^hi^/SULF1^hi^ in PSCs as a distinctive feature in Type-2 diabetes progression, which may contribute to pancreatic islet fibrosis and beta-cell dysfunction. Additionally, our identification of six lncRNAs that potentially interact with activated PSCs sheds light on a possible regulatory lncRNA-mRNA network that could reshape the immune microenvironment in T2D. These findings underscore the importance of PSC activation in T2D and suggest new directions for therapeutic research, particularly in targeting PSC-related pathways. Future studies with expanded datasets and experimental validation are needed to further elucidate the role of PSCs and the identified lncRNAs in T2D pathogenesis.

## Data Availability

The datasets presented in this study can be found in online repositories. The names of the repository/repositories and accession number(s) can be found in the article/**Supplementary Material**.

## Supplementary material

Supplementary Figure 1 Expression status of COL1A2, VCAN and SULF1 across different subgroups.

Supplementary Figure 2 Expression status of COL1A2/VCAN/SULF1 gene panel across different subgroups (***: pvalue < 0.001).

Supplementary Figure 3 Difference of KEGG pathway scores in PSCs between T1D (GSE148073) and T2D samples (GSE86469) (*: pvalue < 0.05).

Supplementary Figure 4 Pearson correlation analysis between Hallmark_pancreas_beta_cells geneset score and Panel/Activation score in 206 T2D samples (GSE76896).
