# Supplementary figures and images for: Identification of a pancreatic stellate cell gene signature and lncRNA interactions associated with type 2 diabetes progression
Source: Front Endocrinol (Lausanne). 2025 Jan 13;15:1532609. doi: 10.3389/fendo.2024.1532609 (PMC11769806; doi:10.3389/fendo.2024.1532609)

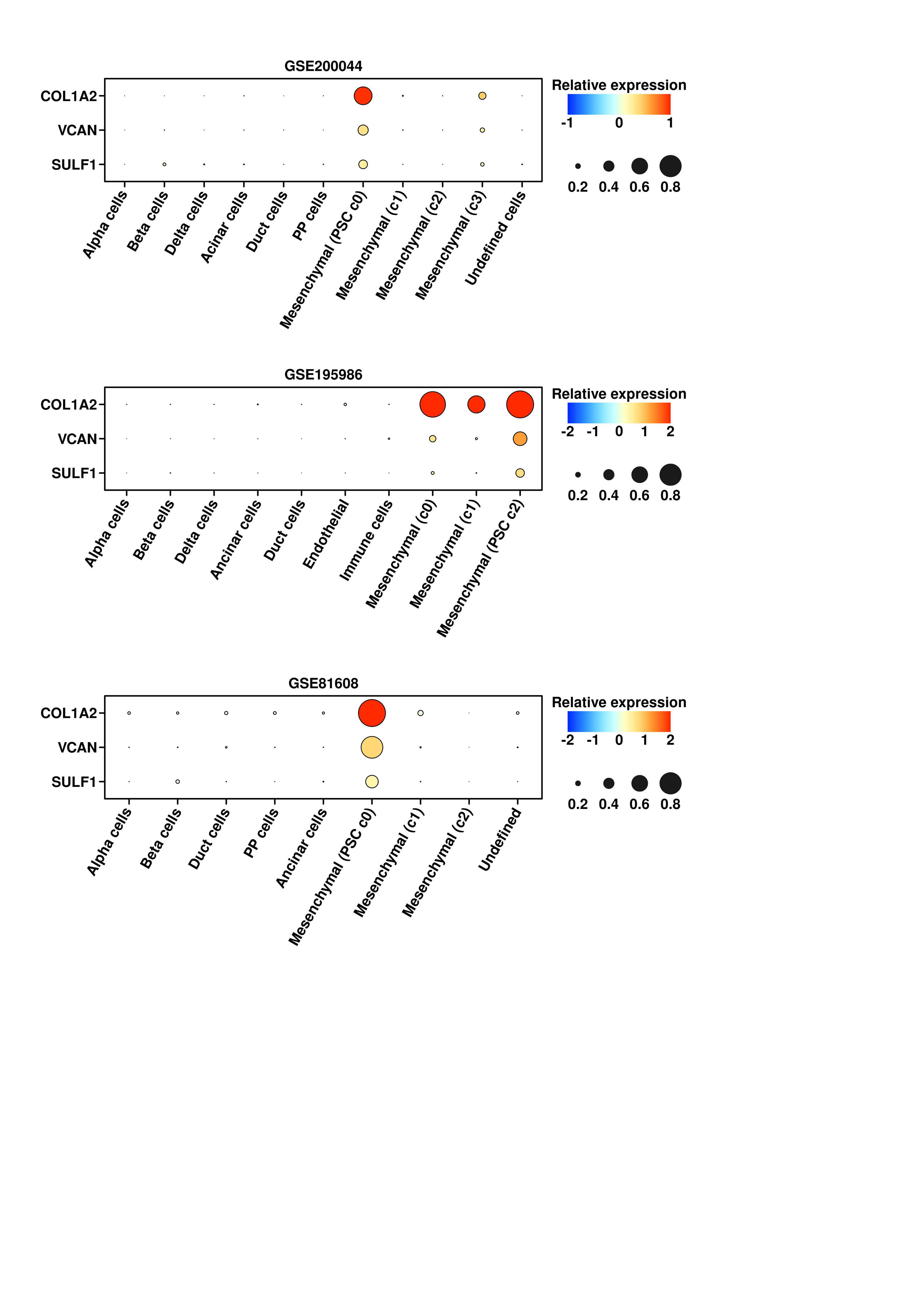

Supplement: Supplementary Figure 1 — Expression status of COL1A2, VCAN and SULF1 across different subgroups. [file Image1.jpeg]

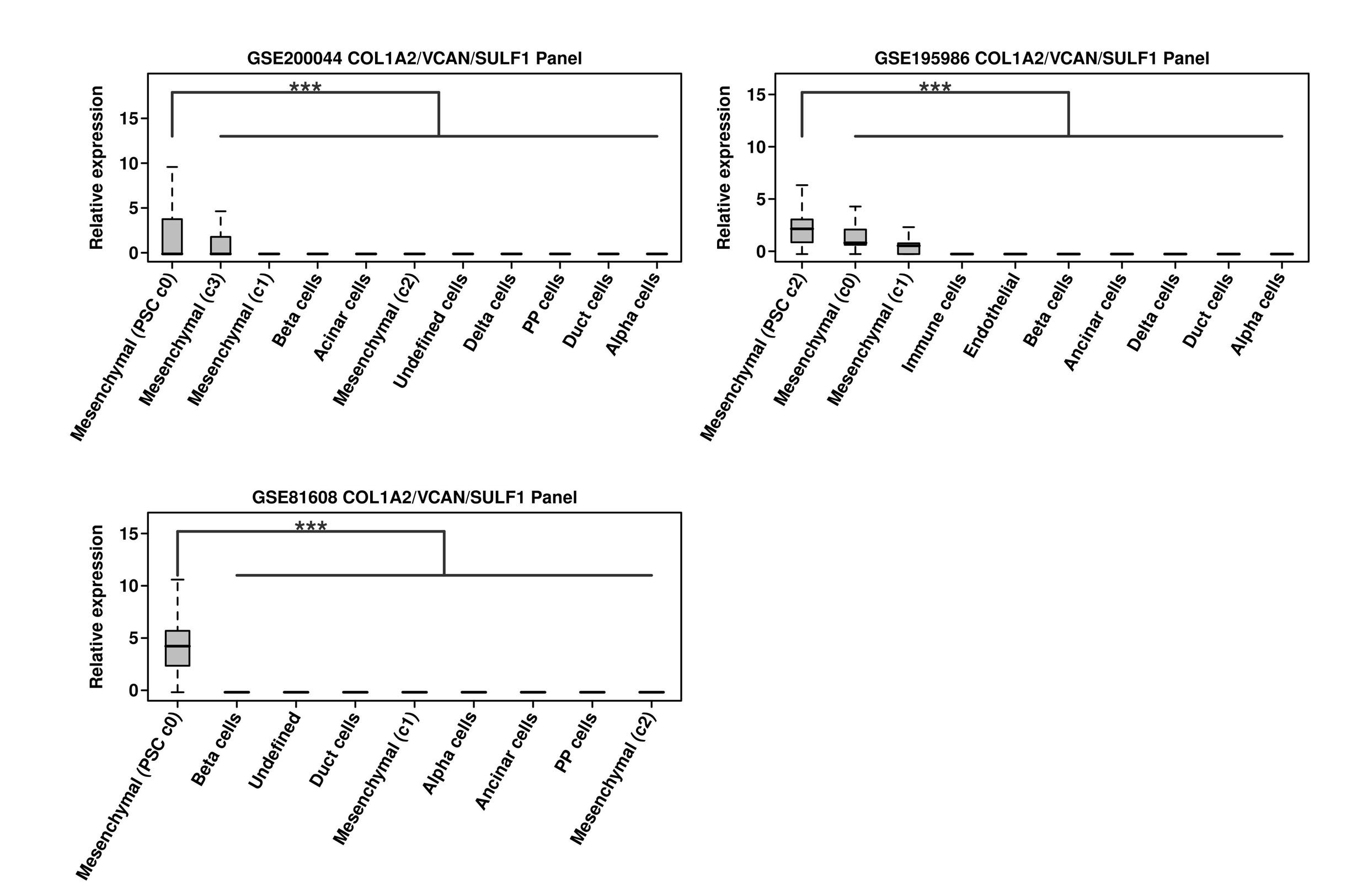

Supplement: Supplementary Figure 2 — Expression status of COL1A2/VCAN/SULF1 gene panel across different subgroups (***: pvalue < 0.001). [file Image2.jpeg]

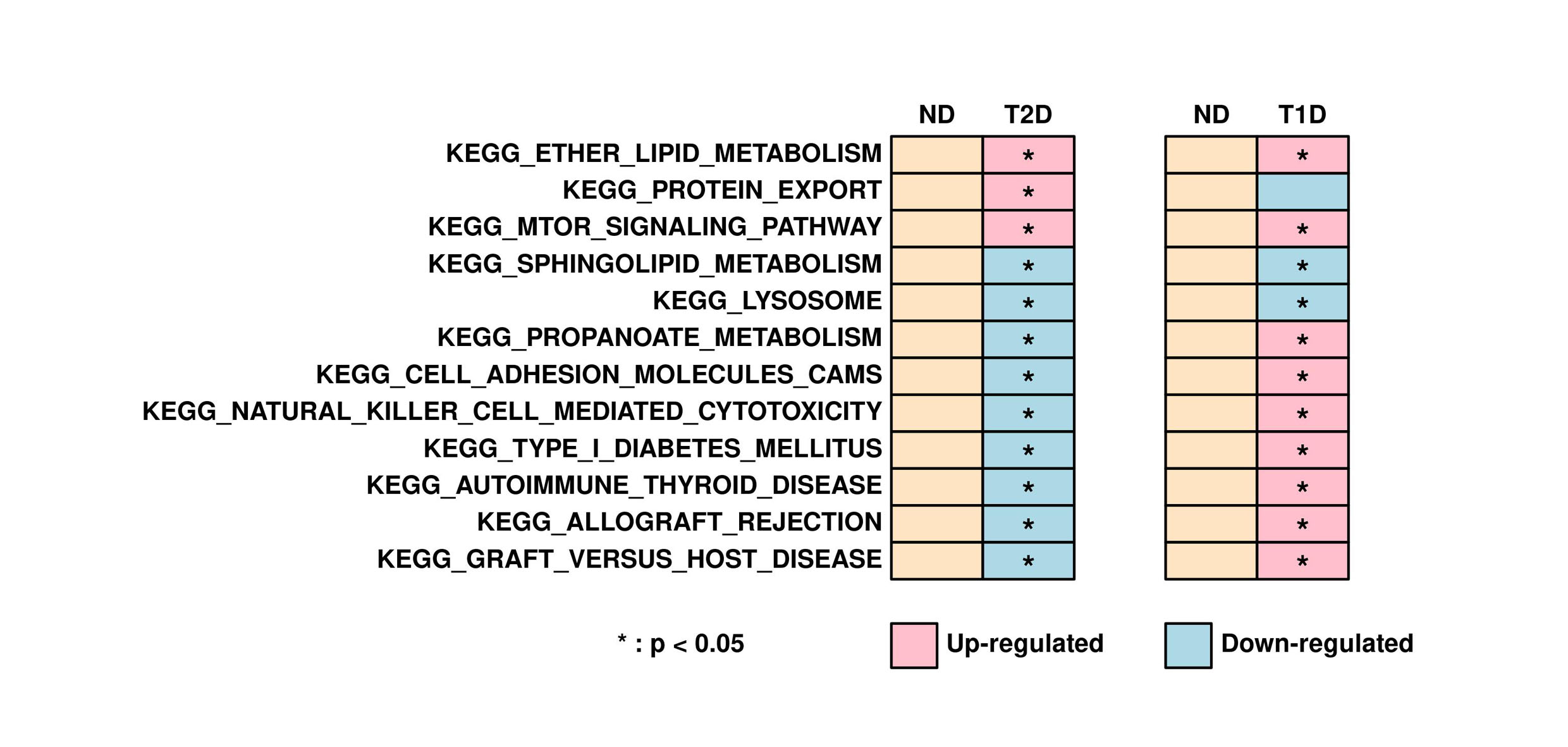

Supplement: Supplementary Figure 3 — Difference of KEGG pathway scores in PSCs between T1D (GSE148073) and T2D samples (GSE86469) (*: pvalue < 0.05). [file Image3.jpeg]

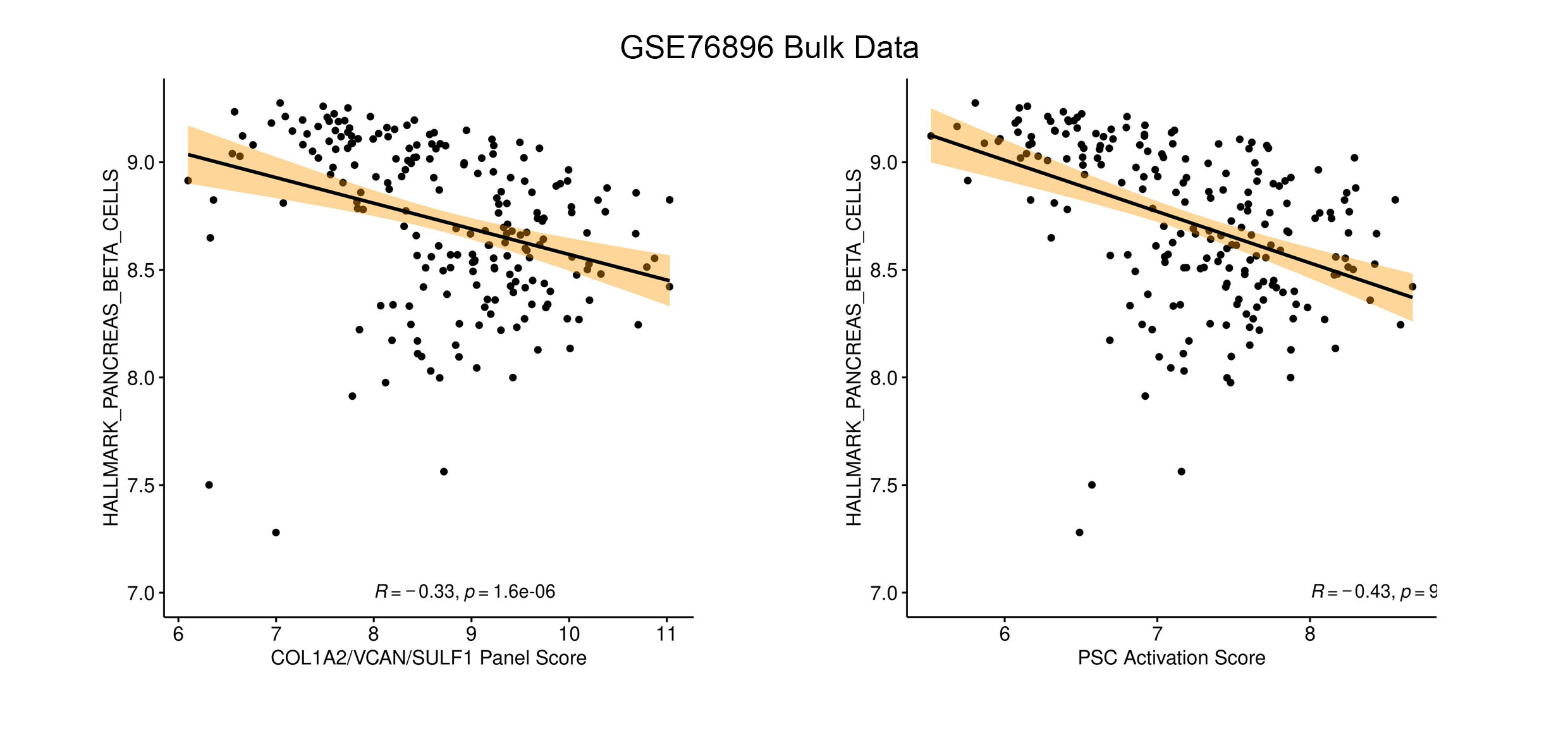

Supplement: Supplementary Figure 4 — Pearson correlation analysis between Hallmark_pancreas_beta_cells geneset score and Panel/Activation score in 206 T2D samples (GSE76896). [file Image4.jpeg]
